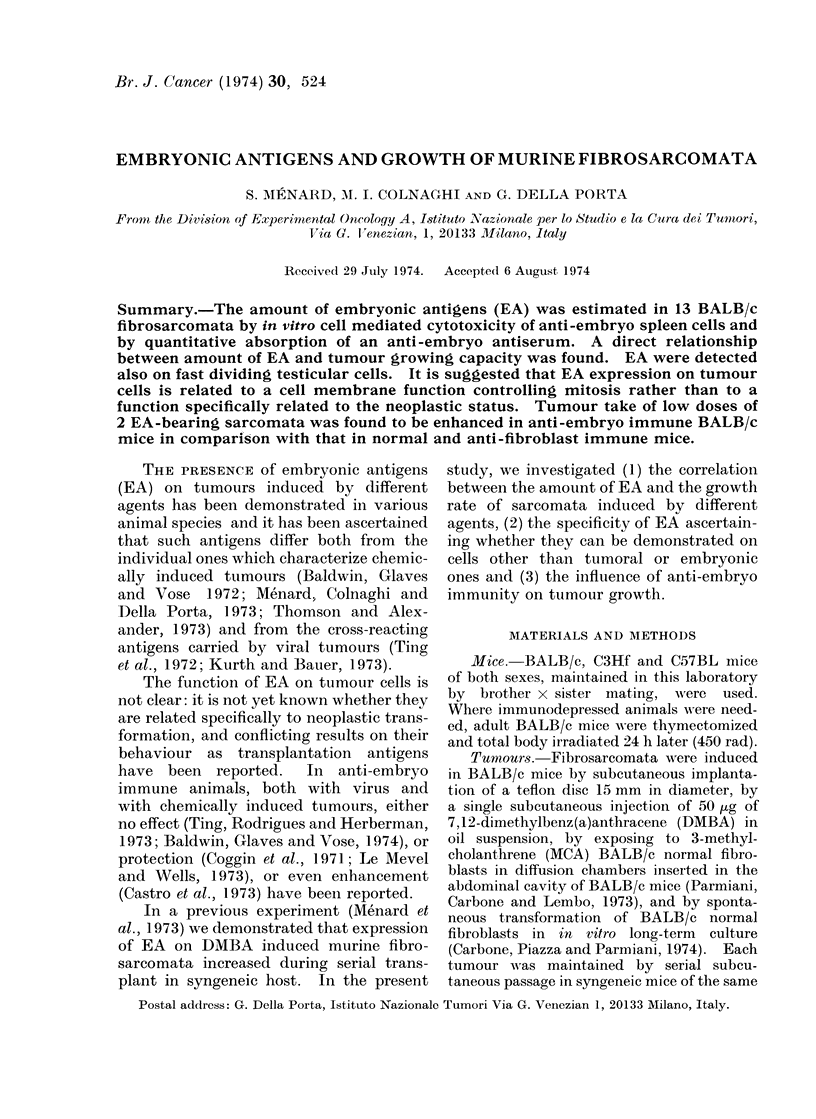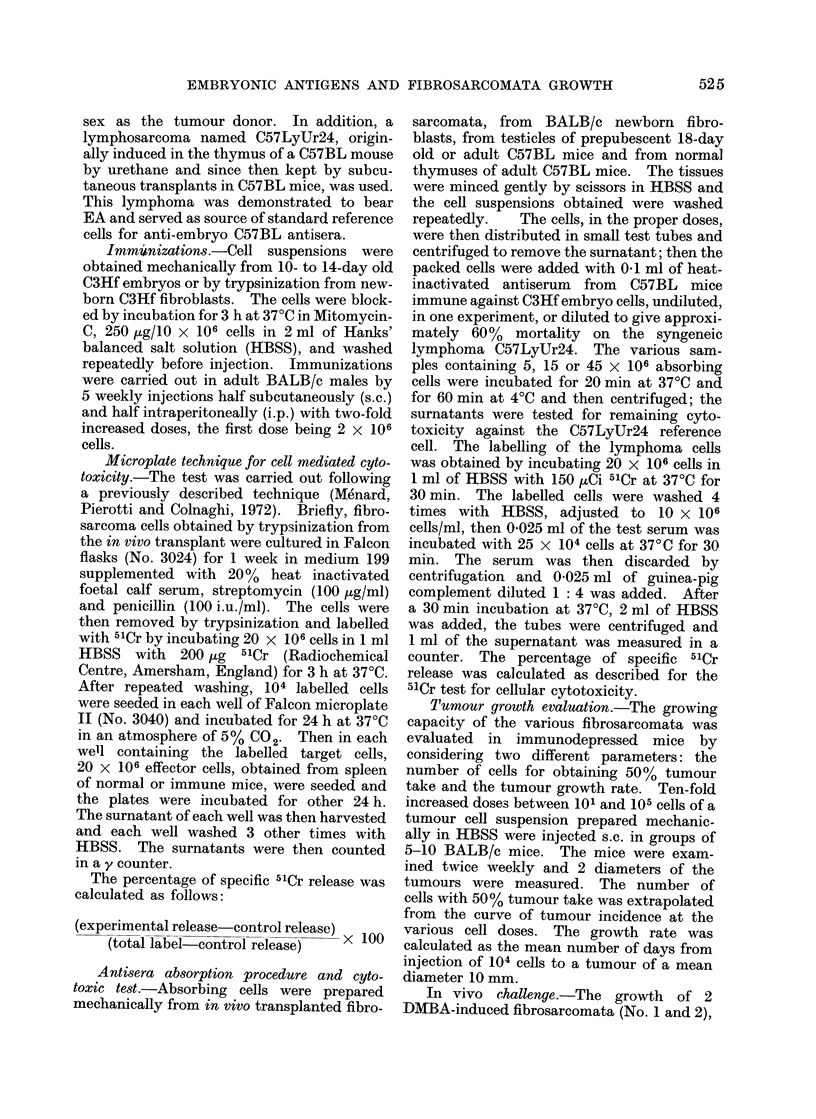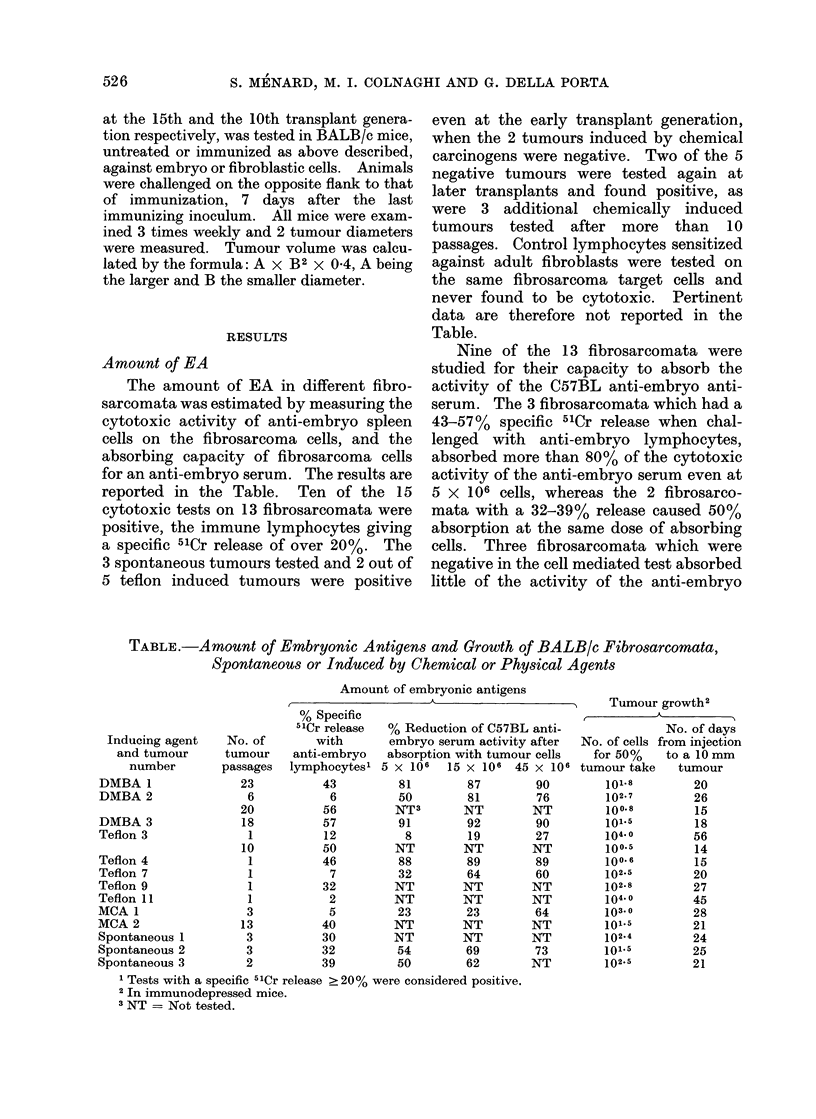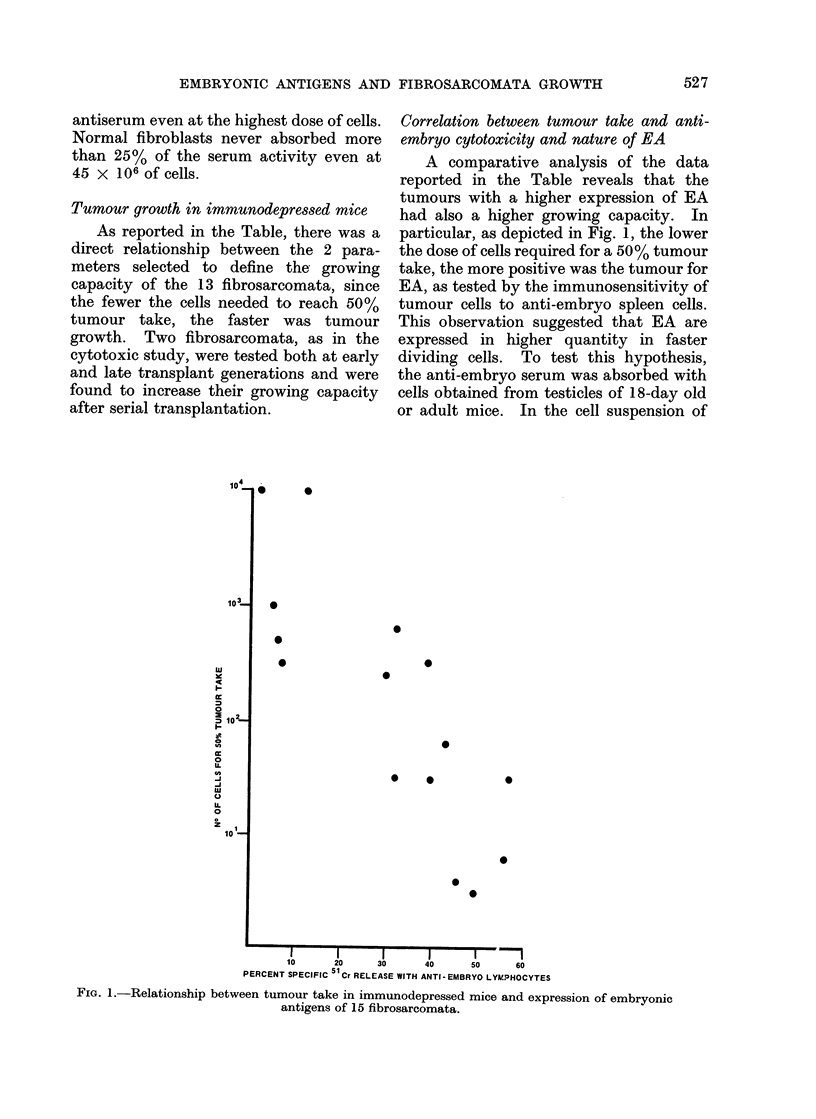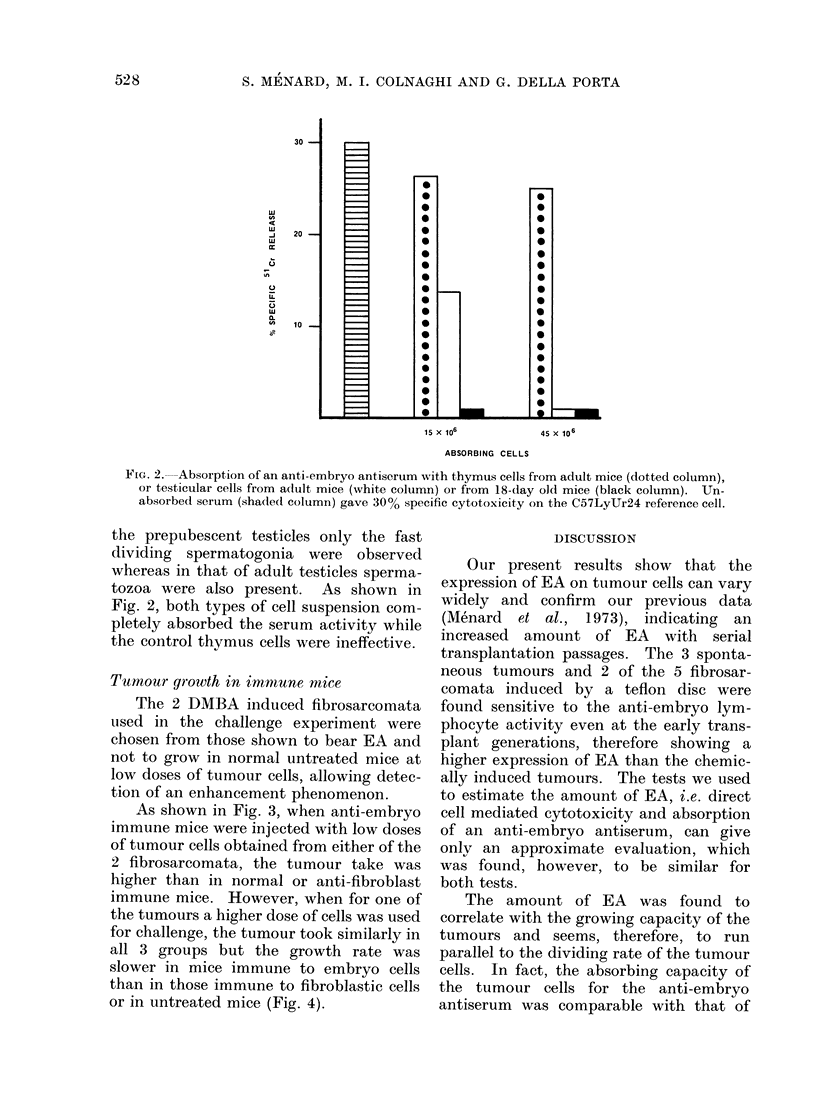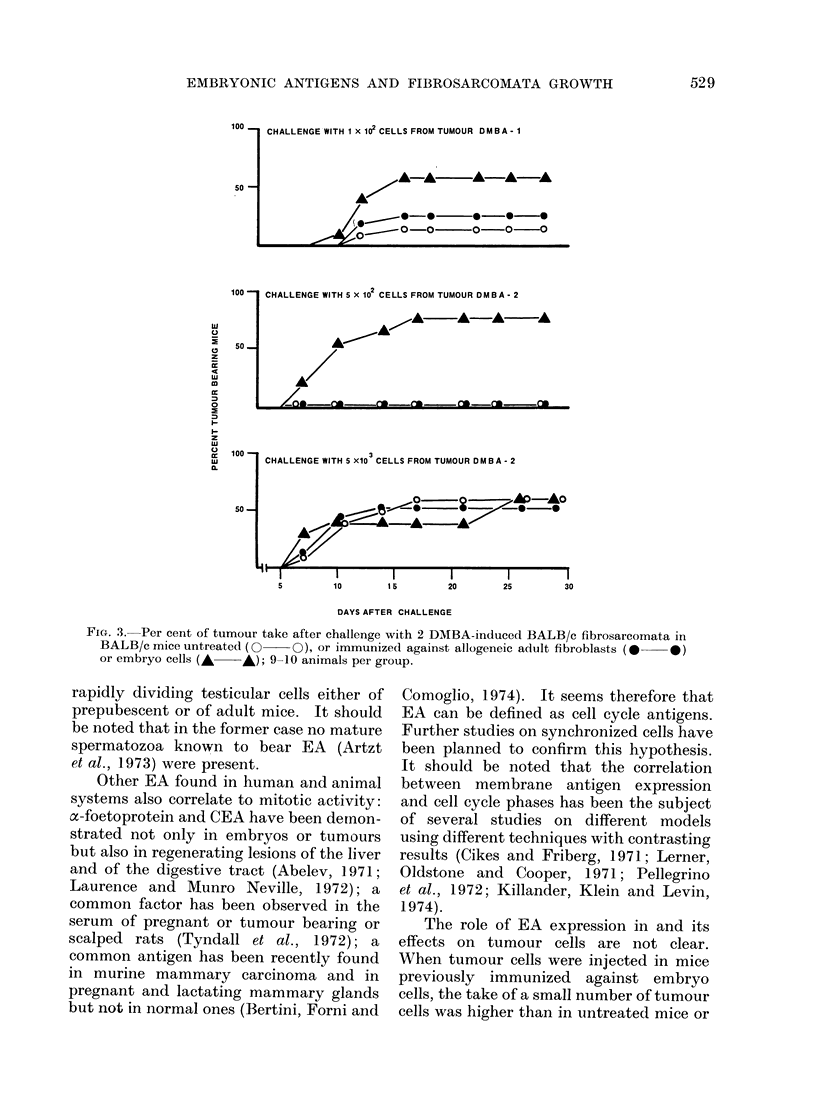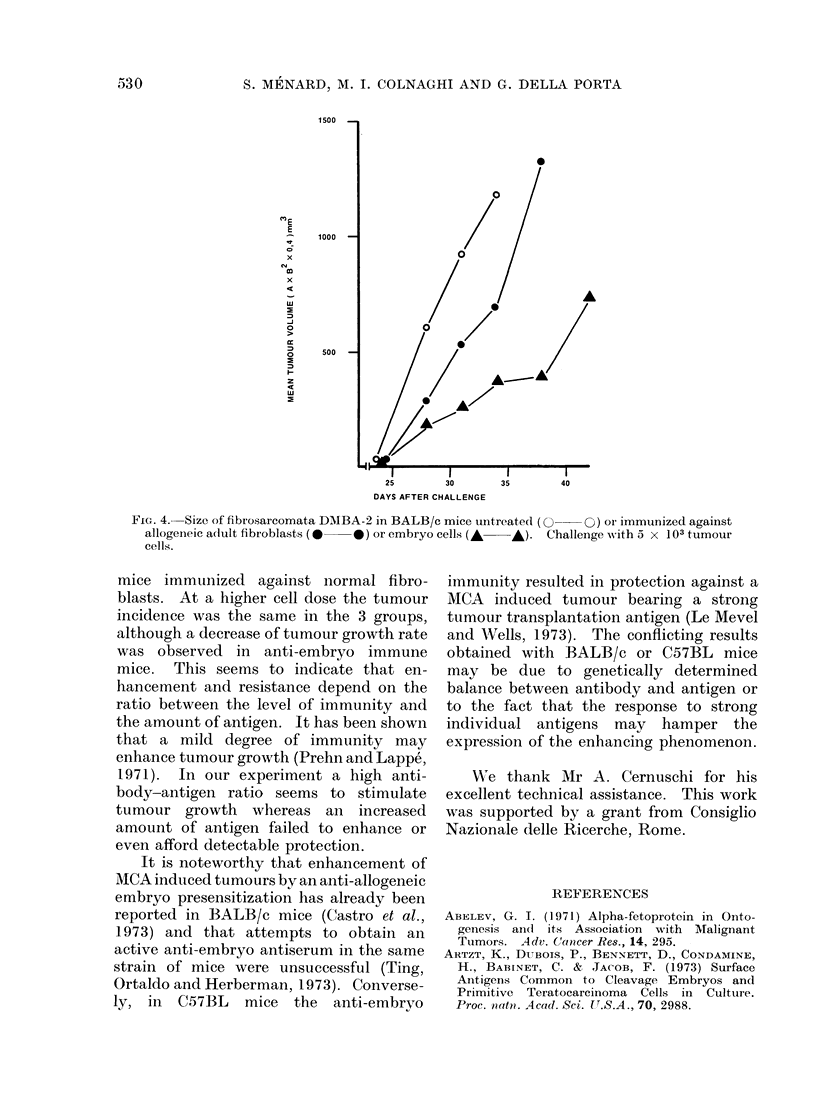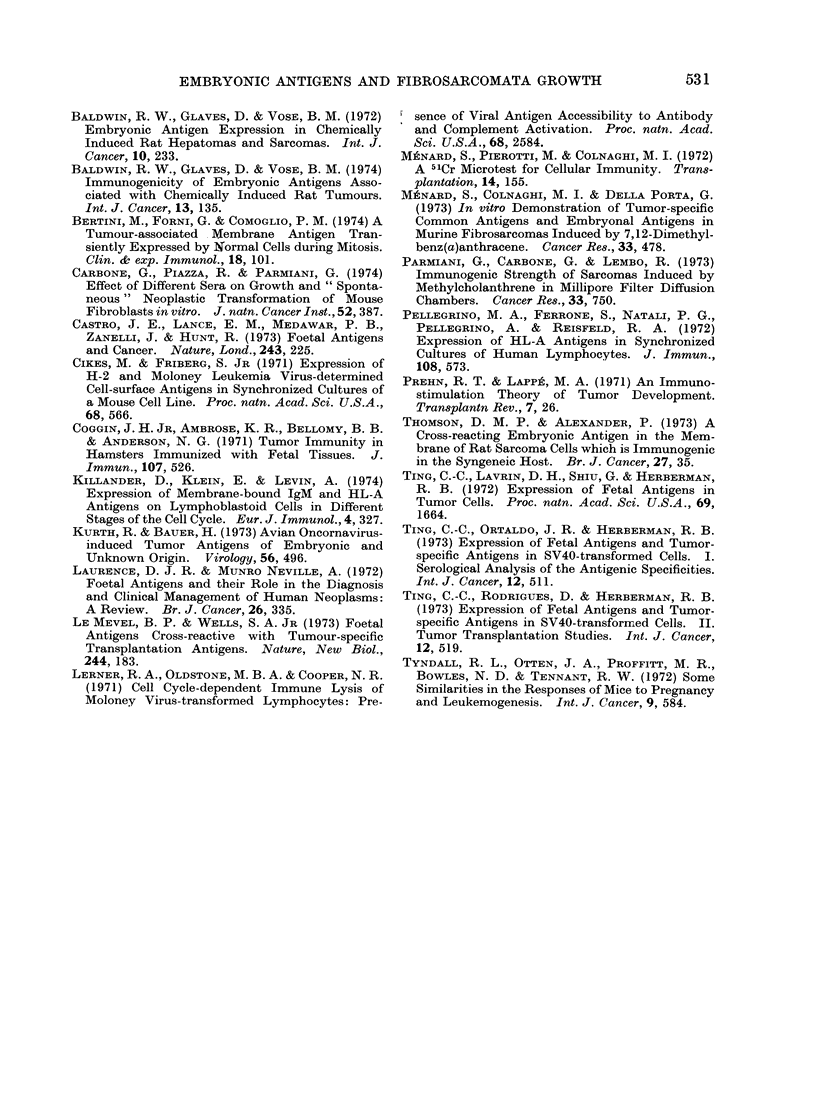# Embryonic Antigens and Growth of Murine Fibrosarcomata

**DOI:** 10.1038/bjc.1974.231

**Published:** 1974-12

**Authors:** S. Ménard, M. L. Colnaghi, G. Della Porta

## Abstract

The amount of embryonic antigens (EA) was estimated in 13 BALB/c fibrosarcomata by *in vitro* cell mediated cytotoxicity of anti-embryo spleen cells and by quantitative absorption of an anti-embryo antiserum. A direct relationship between amount of EA and tumour growing capacity was found. EA were detected also on fast dividing testicular cells. It is suggested that EA expression on tumour cells is related to a cell membrane function controlling mitosis rather than to a function specifically related to the neoplastic status. Tumour take of low doses of 2 EA-bearing sarcomata was found to be enhanced in anti-embryo immune BALB/c mice in comparison with that in normal and anti-fibroblast immune mice.


*Br. J. Cancer* (1974) **30**, 524

### EMBRYONIC ANTIGENS AND GROWTH OF MURINE FIBROSARCOMATA

S. MÉNARD, M. I. COLNAGHI AND G. DELLA PORTA

*From the Division of Experimental Oncology A, Istituto Nazionale per lo Studio e la Cura dei Tumori, Via G. Venezian, 1, 20133 Milano, Italy*

Received 29 July 1974. Accepted 6 August 1974

**Summary.—The amount of embryonic antigens (EA) was estimated in 13 BALB/c fibrosarcomata by *in vitro* cell mediated cytotoxicity of anti-embryo spleen cells and by quantitative absorption of an anti-embryo antiserum. A direct relationship between amount of EA and tumour growing capacity was found. EA were detected also on fast dividing testicular cells. It is suggested that EA expression on tumour cells is related to a cell membrane function controlling mitosis rather than to a function specifically related to the neoplastic status. Tumour take of low doses of 2 EA-bearing sarcomata was found to be enhanced in anti-embryo immune BALB/c mice in comparison with that in normal and anti-fibroblast immune mice.**

THE PRESENCE of embryonic antigens (EA) on tumours induced by different agents has been demonstrated in various animal species and it has been ascertained that such antigens differ both from the individual ones which characterize chemically induced tumours (Baldwin, Glaves and Vose 1972; Ménard, Colnaghi and Della Porta, 1973; Thomson and Alexander, 1973) and from the cross-reacting antigens carried by viral tumours (Ting *et al.*, 1972; Kurth and Bauer, 1973).

The function of EA on tumour cells is not clear: it is not yet known whether they are related specifically to neoplastic transformation, and conflicting results on their behaviour as transplantation antigens have been reported. In anti-embryo immune animals, both with virus and with chemically induced tumours, either no effect (Ting, Rodrigues and Herberman, 1973; Baldwin, Glaves and Vose, 1974), or protection (Coggin *et al.*, 1971; Le Mevel and Wells, 1973), or even enhancement (Castro *et al.*, 1973) have been reported.

In a previous experiment (Ménard *et al.*, 1973) we demonstrated that expression of EA on DMBA induced murine fibrosarcomata increased during serial transplant in syngeneic host. In the present study, we investigated (1) the correlation between the amount of EA and the growth rate of sarcomata induced by different agents, (2) the specificity of EA ascertaining whether they can be demonstrated on cells other than tumoral or embryonic ones and (3) the influence of anti-embryo immunity on tumour growth.

### MATERIALS AND METHODS

*Mice.*—BALB/c, C3Hf and C57BL mice of both sexes, maintained in this laboratory by brother × sister mating, were used. Where immunodepressed animals were needed, adult BALB/c mice were thymectomized and total body irradiated 24 h later (450 rad).

*Tumours.*—Fibrosarcomata were induced in BALB/c mice by subcutaneous implantation of a teflon disc 15 mm in diameter, by a single subcutaneous injection of 50 μg of 7,12-dimethylbenz(a)anthracene (DMBA) in oil suspension, by exposing to 3-methylcholanthrene (MCA) BALB/c normal fibroblasts in diffusion chambers inserted in the abdominal cavity of BALB/c mice (Parmiani, Carbone and Lembo, 1973), and by spontaneous transformation of BALB/c normal fibroblasts in *in vitro* long-term culture (Carbone, Piazza and Parmiani, 1974). Each tumour was maintained by serial subcutaneous passage in syngeneic mice of the same

Postal address: G. Della Porta, Istituto Nazionale Tumori Via G. Venezian 1, 20133 Milano, Italy.

sex as the tumour donor. In addition, a lymphosarcoma named C57LyUr24, originally induced in the thymus of a C57BL mouse by urethane and since then kept by subcutaneous transplants in C57BL mice, was used. This lymphoma was demonstrated to bear EA and served as source of standard reference cells for anti-embryo C57BL antisera.

*Immunizations.*—Cell suspensions were obtained mechanically from 10- to 14-day old C3Hf embryos or by trypsinization from newborn C3Hf fibroblasts. The cells were blocked by incubation for 3 h at 37°C in Mitomycin-C, 250 $\mu$g/10 $\times$ $10^6$ cells in 2 ml of Hanks' balanced salt solution (HBSS), and washed repeatedly before injection. Immunizations were carried out in adult BALB/c males by 5 weekly injections half subcutaneously (s.c.) and half intraperitoneally (i.p.) with two-fold increased doses, the first dose being 2 $\times$ $10^6$ cells.

*Microplate technique for cell mediated cytotoxicity.*—The test was carried out following a previously described technique (Ménard, Pierotti and Colnaghi, 1972). Briefly, fibrosarcoma cells obtained by trypsinization from the *in vivo* transplant were cultured in Falcon flasks (No. 3024) for 1 week in medium 199 supplemented with 20% heat inactivated foetal calf serum, streptomycin (100 $\mu$g/ml) and penicillin (100 i.u./ml). The cells were then removed by trypsinization and labelled with $^{51}$Cr by incubating 20 $\times$ $10^6$ cells in 1 ml HBSS with 200 $\mu$g $^{51}$Cr (Radiochemical Centre, Amersham, England) for 3 h at 37°C. After repeated washing, $10^4$ labelled cells were seeded in each well of Falcon microplate II (No. 3040) and incubated for 24 h at 37°C in an atmosphere of 5% $CO_2$. Then in each well containing the labelled target cells, 20 $\times$ $10^6$ effector cells, obtained from spleen of normal or immune mice, were seeded and the plates were incubated for other 24 h. The surnatant of each well was then harvested and each well washed 3 other times with HBSS. The surnatants were then counted in a $\gamma$ counter.

The percentage of specific $^{51}$Cr release was calculated as follows:

$$\frac{\text{(experimental release—control release)}}{\text{(total label—control release)}} \times 100$$

*Antisera absorption procedure and cytotoxic test.*—Absorbing cells were prepared mechanically from *in vivo* transplanted fibrosarcomata, from BALB/c newborn fibroblasts, from testicles of prepubescent 18-day old or adult C57BL mice and from normal thymuses of adult C57BL mice. The tissues were minced gently by scissors in HBSS and the cell suspensions obtained were washed repeatedly. The cells, in the proper doses, were then distributed in small test tubes and centrifuged to remove the surnatant; then the packed cells were added with 0·1 ml of heat-inactivated antiserum from C57BL mice immune against C3Hf embryo cells, undiluted, in one experiment, or diluted to give approximately 60% mortality on the syngeneic lymphoma C57LyUr24. The various samples containing 5, 15 or 45 $\times$ $10^6$ absorbing cells were incubated for 20 min at 37°C and for 60 min at 4°C and then centrifuged; the surnatants were tested for remaining cytotoxicity against the C57LyUr24 reference cell. The labelling of the lymphoma cells was obtained by incubating 20 $\times$ $10^6$ cells in 1 ml of HBSS with 150 $\mu$Ci $^{51}$Cr at 37°C for 30 min. The labelled cells were washed 4 times with HBSS, adjusted to 10 $\times$ $10^6$ cells/ml, then 0·025 ml of the test serum was incubated with 25 $\times$ $10^4$ cells at 37°C for 30 min. The serum was then discarded by centrifugation and 0·025 ml of guinea-pig complement diluted 1 : 4 was added. After a 30 min incubation at 37°C, 2 ml of HBSS was added, the tubes were centrifuged and 1 ml of the supernatant was measured in a counter. The percentage of specific $^{51}$Cr release was calculated as described for the $^{51}$Cr test for cellular cytotoxicity.

*Tumour growth evaluation.*—The growing capacity of the various fibrosarcomata was evaluated in immunodepressed mice by considering two different parameters: the number of cells for obtaining 50% tumour take and the tumour growth rate. Ten-fold increased doses between $10^1$ and $10^5$ cells of a tumour cell suspension prepared mechanically in HBSS were injected s.c. in groups of 5–10 BALB/c mice. The mice were examined twice weekly and 2 diameters of the tumours were measured. The number of cells with 50% tumour take was extrapolated from the curve of tumour incidence at the various cell doses. The growth rate was calculated as the mean number of days from injection of $10^4$ cells to a tumour of a mean diameter 10 mm.

In vivo *challenge.*—The growth of 2 DMBA-induced fibrosarcomata (No. 1 and 2), at the 15th and the 10th transplant generation respectively, was tested in BALB/c mice, untreated or immunized as above described, against embryo or fibroblastic cells. Animals were challenged on the opposite flank to that of immunization, 7 days after the last immunizing inoculum. All mice were examined 3 times weekly and 2 tumour diameters were measured. Tumour volume was calculated by the formula: $A \times B^2 \times 0{\cdot}4$, A being the larger and B the smaller diameter.

### RESULTS

### *Amount of EA*

The amount of EA in different fibrosarcomata was estimated by measuring the cytotoxic activity of anti-embryo spleen cells on the fibrosarcoma cells, and the absorbing capacity of fibrosarcoma cells for an anti-embryo serum. The results are reported in the Table. Ten of the 15 cytotoxic tests on 13 fibrosarcomata were positive, the immune lymphocytes giving a specific $^{51}Cr$ release of over 20%. The 3 spontaneous tumours tested and 2 out of 5 teflon induced tumours were positive even at the early transplant generation, when the 2 tumours induced by chemical carcinogens were negative. Two of the 5 negative tumours were tested again at later transplants and found positive, as were 3 additional chemically induced tumours tested after more than 10 passages. Control lymphocytes sensitized against adult fibroblasts were tested on the same fibrosarcoma target cells and never found to be cytotoxic. Pertinent data are therefore not reported in the Table.

Nine of the 13 fibrosarcomata were studied for their capacity to absorb the activity of the C57BL anti-embryo antiserum. The 3 fibrosarcomata which had a 43–57% specific $^{51}Cr$ release when challenged with anti-embryo lymphocytes, absorbed more than 80% of the cytotoxic activity of the anti-embryo serum even at $5 \times 10^6$ cells, whereas the 2 fibrosarcomata with a 32–39% release caused 50% absorption at the same dose of absorbing cells. Three fibrosarcomata which were negative in the cell mediated test absorbed little of the activity of the anti-embryo

TABLE.—*Amount of Embryonic Antigens and Growth of BALB/c Fibrosarcomata, Spontaneous or Induced by Chemical or Physical Agents*

| | | Amount of embryonic antigens | | | | Tumour growth[2] | |
|---|---|---|---|---|---|---|---|
| | | | % Reduction of C57BL anti-embryo serum activity after absorption with tumour cells | | | | |
| Inducing agent and tumour number | No. of tumour passages | % Specific $^{51}Cr$ release with anti-embryo lymphocytes[1] | $5 \times 10^6$ | $15 \times 10^6$ | $45 \times 10^6$ | No. of cells for 50% tumour take | No. of days from injection to a 10 mm tumour |
| DMBA 1 | 23 | 43 | 81 | 87 | 90 | $10^{1 \cdot 8}$ | 20 |
| DMBA 2 | 6 | 6 | 50 | 81 | 76 | $10^{2 \cdot 7}$ | 26 |
| | 20 | 56 | NT[3] | NT | NT | $10^{0 \cdot 8}$ | 15 |
| DMBA 3 | 18 | 57 | 91 | 92 | 90 | $10^{1 \cdot 5}$ | 18 |
| Teflon 3 | 1 | 12 | 8 | 19 | 27 | $10^{4 \cdot 0}$ | 56 |
| | 10 | 50 | NT | NT | NT | $10^{0 \cdot 5}$ | 14 |
| Teflon 4 | 1 | 46 | 88 | 89 | 89 | $10^{0 \cdot 6}$ | 15 |
| Teflon 7 | 1 | 7 | 32 | 64 | 60 | $10^{2 \cdot 5}$ | 20 |
| Teflon 9 | 1 | 32 | NT | NT | NT | $10^{2 \cdot 8}$ | 27 |
| Teflon 11 | 1 | 2 | NT | NT | NT | $10^{4 \cdot 0}$ | 45 |
| MCA 1 | 3 | 5 | 23 | 23 | 64 | $10^{3 \cdot 0}$ | 28 |
| MCA 2 | 13 | 40 | NT | NT | NT | $10^{1 \cdot 5}$ | 21 |
| Spontaneous 1 | 3 | 30 | NT | NT | NT | $10^{2 \cdot 4}$ | 24 |
| Spontaneous 2 | 3 | 32 | 54 | 69 | 73 | $10^{1 \cdot 5}$ | 25 |
| Spontaneous 3 | 2 | 39 | 50 | 62 | NT | $10^{2 \cdot 5}$ | 21 |

[1] Tests with a specific $^{51}Cr$ release $\geq 20\%$ were considered positive.
[2] In immunodepressed mice.
[3] NT = Not tested.

antiserum even at the highest dose of cells. Normal fibroblasts never absorbed more than 25% of the serum activity even at $45 \times 10^6$ of cells.

*Tumour growth in immunodepressed mice*

As reported in the Table, there was a direct relationship between the 2 parameters selected to define the growing capacity of the 13 fibrosarcomata, since the fewer the cells needed to reach 50% tumour take, the faster was tumour growth. Two fibrosarcomata, as in the cytotoxic study, were tested both at early and late transplant generations and were found to increase their growing capacity after serial transplantation.

*Correlation between tumour take and anti-embryo cytotoxicity and nature of EA*

A comparative analysis of the data reported in the Table reveals that the tumours with a higher expression of EA had also a higher growing capacity. In particular, as depicted in Fig. 1, the lower the dose of cells required for a 50% tumour take, the more positive was the tumour for EA, as tested by the immunosensitivity of tumour cells to anti-embryo spleen cells. This observation suggested that EA are expressed in higher quantity in faster dividing cells. To test this hypothesis, the anti-embryo serum was absorbed with cells obtained from testicles of 18-day old or adult mice. In the cell suspension of

FIG. 1.—Relationship between tumour take in immunodepressed mice and expression of embryonic antigens of 15 fibrosarcomata.

FIG. 2.—Absorption of an anti-embryo antiserum with thymus cells from adult mice (dotted column), or testicular cells from adult mice (white column) or from 18-day old mice (black column). Unabsorbed serum (shaded column) gave 30% specific cytotoxicity on the C57LyUr24 reference cell.

the prepubescent testicles only the fast dividing spermatogonia were observed whereas in that of adult testicles spermatozoa were also present. As shown in Fig. 2, both types of cell suspension completely absorbed the serum activity while the control thymus cells were ineffective.

### *Tumour growth in immune mice*

The 2 DMBA induced fibrosarcomata used in the challenge experiment were chosen from those shown to bear EA and not to grow in normal untreated mice at low doses of tumour cells, allowing detection of an enhancement phenomenon.

As shown in Fig. 3, when anti-embryo immune mice were injected with low doses of tumour cells obtained from either of the 2 fibrosarcomata, the tumour take was higher than in normal or anti-fibroblast immune mice. However, when for one of the tumours a higher dose of cells was used for challenge, the tumour took similarly in all 3 groups but the growth rate was slower in mice immune to embryo cells than in those immune to fibroblastic cells or in untreated mice (Fig. 4).

### DISCUSSION

Our present results show that the expression of EA on tumour cells can vary widely and confirm our previous data (Ménard *et al.*, 1973), indicating an increased amount of EA with serial transplantation passages. The 3 spontaneous tumours and 2 of the 5 fibrosarcomata induced by a teflon disc were found sensitive to the anti-embryo lymphocyte activity even at the early transplant generations, therefore showing a higher expression of EA than the chemically induced tumours. The tests we used to estimate the amount of EA, *i.e.* direct cell mediated cytotoxicity and absorption of an anti-embryo antiserum, can give only an approximate evaluation, which was found, however, to be similar for both tests.

The amount of EA was found to correlate with the growing capacity of the tumours and seems, therefore, to run parallel to the dividing rate of the tumour cells. In fact, the absorbing capacity of the tumour cells for the anti-embryo antiserum was comparable with that of

FIG. 3.—Per cent of tumour take after challenge with 2 DMBA-induced BALB/c fibrosarcomata in BALB/c mice untreated (○——○), or immunized against allogeneic adult fibroblasts (●——●) or embryo cells (▲——▲); 9–10 animals per group.

rapidly dividing testicular cells either of prepubescent or of adult mice. It should be noted that in the former case no mature spermatozoa known to bear EA (Artzt *et al.*, 1973) were present.

Other EA found in human and animal systems also correlate to mitotic activity: $\alpha$-foetoprotein and CEA have been demonstrated not only in embryos or tumours but also in regenerating lesions of the liver and of the digestive tract (Abelev, 1971; Laurence and Munro Neville, 1972); a common factor has been observed in the serum of pregnant or tumour bearing or scalped rats (Tyndall *et al.*, 1972); a common antigen has been recently found in murine mammary carcinoma and in pregnant and lactating mammary glands but not in normal ones (Bertini, Forni and Comoglio, 1974). It seems therefore that EA can be defined as cell cycle antigens. Further studies on synchronized cells have been planned to confirm this hypothesis. It should be noted that the correlation between membrane antigen expression and cell cycle phases has been the subject of several studies on different models using different techniques with contrasting results (Cikes and Friberg, 1971; Lerner, Oldstone and Cooper, 1971; Pellegrino *et al.*, 1972; Killander, Klein and Levin, 1974).

The role of EA expression in and its effects on tumour cells are not clear. When tumour cells were injected in mice previously immunized against embryo cells, the take of a small number of tumour cells was higher than in untreated mice or

FIG. 4.—Size of fibrosarcomata DMBA-2 in BALB/c mice untreated (○——○) or immunized against allogeneic adult fibroblasts (●——●) or embryo cells (▲——▲). Challenge with 5 × $10^3$ tumour cells.

mice immunized against normal fibroblasts. At a higher cell dose the tumour incidence was the same in the 3 groups, although a decrease of tumour growth rate was observed in anti-embryo immune mice. This seems to indicate that enhancement and resistance depend on the ratio between the level of immunity and the amount of antigen. It has been shown that a mild degree of immunity may enhance tumour growth (Prehn and Lappé, 1971). In our experiment a high antibody–antigen ratio seems to stimulate tumour growth whereas an increased amount of antigen failed to enhance or even afford detectable protection.

It is noteworthy that enhancement of MCA induced tumours by an anti-allogeneic embryo presensitization has already been reported in BALB/c mice (Castro *et al.*, 1973) and that attempts to obtain an active anti-embryo antiserum in the same strain of mice were unsuccessful (Ting, Ortaldo and Herberman, 1973). Conversely, in C57BL mice the anti-embryo immunity resulted in protection against a MCA induced tumour bearing a strong tumour transplantation antigen (Le Mevel and Wells, 1973). The conflicting results obtained with BALB/c or C57BL mice may be due to genetically determined balance between antibody and antigen or to the fact that the response to strong individual antigens may hamper the expression of the enhancing phenomenon.

We thank Mr A. Cernuschi for his excellent technical assistance. This work was supported by a grant from Consiglio Nazionale delle Ricerche, Rome.

### REFERENCES

ABELEV, G. I. (1971) Alpha-fetoprotein in Ontogenesis and its Association with Malignant Tumors. *Adv. Cancer Res.*, **14**, 295.

ARTZT, K., DUBOIS, P., BENNETT, D., CONDAMINE, H., BABINET, C. & JACOB, F. (1973) Surface Antigens Common to Cleavage Embryos and Primitive Teratocarcinoma Cells in Culture. *Proc. natn. Acad. Sci. U.S.A.*, **70**, 2988.

BALDWIN, R. W., GLAVES, D. & VOSE, B. M. (1972) Embryonic Antigen Expression in Chemically Induced Rat Hepatomas and Sarcomas. *Int. J. Cancer*, **10**, 233.

BALDWIN, R. W., GLAVES, D. & VOSE, B. M. (1974) Immunogenicity of Embryonic Antigens Associated with Chemically Induced Rat Tumours. *Int. J. Cancer*, **13**, 135.

BERTINI, M., FORNI, G. & COMOGLIO, P. M. (1974) A Tumour-associated Membrane Antigen Transiently Expressed by Normal Cells during Mitosis. *Clin. & exp. Immunol.*, **18**, 101.

CARBONE, G., PIAZZA, R. & PARMIANI, G. (1974) Effect of Different Sera on Growth and " Spontaneous " Neoplastic Transformation of Mouse Fibroblasts *in vitro*. *J. natn. Cancer Inst.*, **52**, 387.

CASTRO, J. E., LANCE, E. M., MEDAWAR, P. B., ZANELLI, J. & HUNT, R. (1973) Foetal Antigens and Cancer. *Nature, Lond.*, **243**, 225.

CIKES, M. & FRIBERG, S. JR (1971) Expression of H-2 and Moloney Leukemia Virus-determined Cell-surface Antigens in Synchronized Cultures of a Mouse Cell Line. *Proc. natn. Acad. Sci. U.S.A.*, **68**, 566.

COGGIN, J. H. JR, AMBROSE, K. R., BELLOMY, B. B. & ANDERSON, N. G. (1971) Tumor Immunity in Hamsters Immunized with Fetal Tissues. *J. Immun.*, **107**, 526.

KILLANDER, D., KLEIN, E. & LEVIN, A. (1974) Expression of Membrane-bound IgM and HL-A Antigens on Lymphoblastoid Cells in Different Stages of the Cell Cycle. *Eur. J. Immunol.*, **4**, 327.

KURTH, R. & BAUER, H. (1973) Avian Oncornavirus-induced Tumor Antigens of Embryonic and Unknown Origin. *Virology*, **56**, 496.

LAURENCE, D. J. R. & MUNRO NEVILLE, A. (1972) Foetal Antigens and their Role in the Diagnosis and Clinical Management of Human Neoplasms: A Review. *Br. J. Cancer*, **26**, 335.

LE MEVEL, B. P. & WELLS, S. A. JR (1973) Foetal Antigens Cross-reactive with Tumour-specific Transplantation Antigens. *Nature, New Biol.*, **244**, 183.

LERNER, R. A., OLDSTONE, M. B. A. & COOPER, N. R. (1971) Cell Cycle-dependent Immune Lysis of Moloney Virus-transformed Lymphocytes: Presence of Viral Antigen Accessibility to Antibody and Complement Activation. *Proc. natn. Acad. Sci. U.S.A.*, **68**, 2584.

MÉNARD, S., PIEROTTI, M. & COLNAGHI, M. I. (1972) A $^{51}$Cr Microtest for Cellular Immunity. *Transplantation*, **14**, 155.

MÉNARD, S., COLNAGHI, M. I. & DELLA PORTA, G. (1973) *In vitro* Demonstration of Tumor-specific Common Antigens and Embryonal Antigens in Murine Fibrosarcomas Induced by 7,12-Dimethylbenz(*a*)anthracene. *Cancer Res.*, **33**, 478.

PARMIANI, G., CARBONE, G. & LEMBO, R. (1973) Immunogenic Strength of Sarcomas Induced by Methylcholanthrene in Millipore Filter Diffusion Chambers. *Cancer Res.*, **33**, 750.

PELLEGRINO, M. A., FERRONE, S., NATALI, P. G., PELLEGRINO, A. & REISFELD, R. A. (1972) Expression of HL-A Antigens in Synchronized Cultures of Human Lymphocytes. *J. Immun.*, **108**, 573.

PREHN, R. T. & LAPPÉ, M. A. (1971) An Immunostimulation Theory of Tumor Development. *Transplantn Rev.*, **7**, 26.

THOMSON, D. M. P. & ALEXANDER, P. (1973) A Cross-reacting Embryonic Antigen in the Membrane of Rat Sarcoma Cells which is Immunogenic in the Syngeneic Host. *Br. J. Cancer*, **27**, 35.

TING, C.-C., LAVRIN, D. H., SHIU, G. & HERBERMAN, R. B. (1972) Expression of Fetal Antigens in Tumor Cells. *Proc. natn. Acad. Sci. U.S.A.*, **69**, 1664.

TING, C.-C., ORTALDO, J. R. & HERBERMAN, R. B. (1973) Expression of Fetal Antigens and Tumor-specific Antigens in SV40-transformed Cells. I. Serological Analysis of the Antigenic Specificities. *Int. J. Cancer*, **12**, 511.

TING, C.-C., RODRIGUES, D. & HERBERMAN, R. B. (1973) Expression of Fetal Antigens and Tumor-specific Antigens in SV40-transformed Cells. II. Tumor Transplantation Studies. *Int. J. Cancer*, **12**, 519.

TYNDALL, R. L., OTTEN, J. A., PROFFITT, M. R., BOWLES, N. D. & TENNANT, R. W. (1972) Some Similarities in the Responses of Mice to Pregnancy and Leukemogenesis. *Int. J. Cancer*, **9**, 584.